# Improvement of Guideline Adherence After the Implementation of an Antibiotic Stewardship Program in a Secondary Care Pediatric Hospital

**DOI:** 10.3389/fped.2019.00478

**Published:** 2019-11-13

**Authors:** Jakob Metz, Philipp Oehler, Manuela Burggraf, Stefan Burdach, Uta Behrends, Nikolaus Rieber

**Affiliations:** ^1^Klinik für Kinder- und Jugendmedizin, München Klinik Schwabing und Harlaching; and Department of Pediatrics, Technical University of Munich School of Medicine, Munich, Germany; ^2^German Center for Infection Research (DZIF), Partner Site Munich, Munich, Germany; ^3^Children's Hospital, University of Tübingen, Tübingen, Germany

**Keywords:** antibiotic stewardship, pediatrics, ASP, antimicrobial stewardship, multidrug-resistance, pediatric hospital, secondary care

## Abstract

**Introduction:** The accelerating threat of multidrug-resistant bacteria (MRB) forces health care providers to use antibiotics more rationally. Antibiotic stewardship programs (ASP) are a proven and safe way to achieve that goal. They have been comprehensively studied in adults but data from secondary care pediatric hospitals are lacking.

**Material and Methods:** In our study an ASP with standard operating procedures (SOPs), audits, a weekly ward round with experts in pediatric infectious diseases and an antibiotic pocket-card for selected infectious diseases was established in July 2017 in a Munich municipal secondary care children's hospital. All antibiotic prescriptions on general pediatric wards were reviewed each in the first quarter of 2017 and 2018. The primary outcome was adherence to treatment guidelines. Secondary outcomes were substance consumption, duration of therapy and death.

**Results:** After the ASP was implemented guideline adherence increased significantly from 33 to 63%. The consumption of cephalosporins decreased significantly (−60%), whereas aminopenicillin use increased accordingly (+120%). Neither in the pre- nor in the post-intervention group deaths occurred.

**Discussion:** Data on ASP in pediatric secondary care hospitals are scarce. Most previous studies have been performed at tertiary care/university children's hospitals. We demonstrate a significant improvement in guideline adherence regarding antibiotic treatments after the implementation of an ASP. Cephalosporin consumption decreased which might be relevant for the selection of MRB (e.g., vancomycin-resistant enterococci). Results are limited by the single-center design and the short observation period. The study encourages the implementation of ASPs in secondary care children's hospitals.

## Introduction

The number of newly approved antibiotic substances is constantly declining over the last decades. On the other hand, infections with multidrug-resistant bacteria (MRB) are a leading global health care threat (1, 2). The implementation of antibiotic stewardship programs (ASPs) has been proven effective to reduce the burden of infections with MRB such as extended-spectrum beta-lactamase (ESBL)-producing enterobacteriaceae, vancomycin-resistant enterococcus (VRE), and methicillin-resistant *Staphylococcus aureus* (MRSA) (3, 4). A widely accepted goal in ASPs is the reduction of cephalosporin consumption as a major factor for the selection of MRB. *Enterococcus* spp. display a natural resistance to cephalosporins leading to a selection advantage for vancomycin-susceptible and vancomycin-resistant enterococci (VRE) (5, 6). Further beneficial impacts of ASPs include avoiding adverse effects to unnecessary antibiotic therapies and the overall cost savings for the health systems (7, 8).

The body of evidence regarding ASPs mainly consists of studies in adult patients, but data from pediatric settings have steadily increased over the last years (4). There are some special challenges, which need to be considered when dealing with ASPs in children and infants. In adults the consumption of medications can be measured with so called *defined daily doses* (DDD). The DDDs are standardized and the extent of antibiotic usage can be determined by requesting the amount of orders in the clinical pharmacy. This amount can then be related to the *patient days* (PD). One PD is 1 day of inpatient treatment of one patient in the hospital. Together this represents an easy way to monitor the amount of antibiotic usage. In children and infants the drug dosage is routinely adjusted to the body weight. To measure the consumption of antibiotics with DDD is therefore not feasible. The best solution is to measure *days of therapy* (DOT). One DOT means that a patient is treated for 1 day with one specific drug. Since sometimes more than one drug is used simultaneously DOTs can be overlapping. This is the reason why the total duration of therapy should also be evaluated using the item *length of therapy* (LOT) (9).

In a Cochrane systematic review in adults and children one of the outcome variables was the adherence to treatment guidelines and how ASPs affect it. The authors found 29 randomized controlled trials (RCT) with a total of 23,394 patients. They demonstrated an increase of guideline adherence from 43 to 58% after ASPs had been launched. The mean duration of antibiotic treatment decreased from eleven to 9 days (14 RCTs, 3,318 participants). The interventions were deemed safe by the authors as mortality did not change upon ASP implementation (4). In children data are limited but few studies also reveal positive results for ASPs (10–12). Smith et al. demonstrate a reduction of the duration of therapy, of false prescriptions and of costs after the implementation of ASPs in pediatrics (10). A German group determined estimated cost savings of € 330,000 per year in a tertiary care pediatric university hospital (13). In the same hospital the consumption of antibiotics was examined after the ASP had been launched. DOTs of cephalosporins and LOTs decreased significantly, whereas guideline conform treatment in patients with pneumonia increased from 39.5 to 93.8%. The total amounts of antibiotic treatments remained stable (8). So far, no pediatric study showed adverse effects of such interventions. Data from primary care and secondary care/municipal hospitals are scarce. Therefore, we set out a study to determine effects of an ASP in a secondary care municipal hospital in Munich, Germany. We show here that implementing an ASP is successful in a secondary care pediatric hospital with regard to guideline adherence and reduction of cephalosporin consumption.

## Materials and Methods

We investigated antibiotic prescriptions in a municipal secondary care pediatric hospital in Munich, Germany (*Kinderklinik München Harlaching*). The hospital consists of three general pediatric wards and a neonatal intensive care unit (NICU). All antibiotic prescriptions for inpatients were collected for the first quarter of 2017 and 2018. We excluded the NICU and antibiotic prophylaxis [e.g., in recurrent urinary tract infections (UTIs)] from deductive statistics. Data were collected from patient charts and discharge reports. In cases in which a further antibiotic treatment after discharge had been recommended we assumed it accomplished as recommended in the reports. This led to consideration of the entire antibiotic treatment of each patient.

In July 2017 anASP was implemented. It consisted of a prospective audit with feedback by experts in pediatric infectious diseases, in-house treatment guidelines as *standard operating procedures* (SOPs) for selected infectious diseases and a pocket-card, which summarizes the key information of the SOPs. SOPs were established for acute otitis media (AOM), pneumonia, meningitis, tonsillitis, and UTI.

The primary outcome of our study was the adherence to treatment guidelines before vs. after implementation of the ASP. In the pre-interventional group guideline adherence was defined as treatment according to the textbook published by the German Pediatric Infectious Disease Society (*Deutsche Gesellschaft für Pädiatrische Infektiologie*, DGPI e.V.) (14). In the post-intervention group, the newly launched SOPs were used to define guideline adherence. Table 1 summarizes the pharmacological key recommendations underlying our study.

**Table 1:** Recommendations by the DGPI (14) (shortened) vs. new in-house SOPs.

	**DGPI 2013** **(****14****)**		**SOP**	
Pneumonia^*^	Aminopenicillin (±Macrolid)	Abscessing cases: Aminopenicillin + BLI (±Macrolid)	Non-severe cases: Aminopenicillin	Severe cases: Aminopenicillin+BLI (±Macrolid)
Pyelonephritis	Age <6 months: Ceftazidime + Ampicillin^**^	Age > 6 months: Cephalosporin 3rd generation	Age <12 months: Ampicillin + Ceftazidime	Age > 12 months: Cefpodoxime or Cefotaxime
Tonsillitis	Penicillin		Penicillin	

* Above 3 months of age

** *where appropriate until 12 months*.

Secondary outcomes were the usage of specific classes of antibiotic drugs. They were measured in DOT. Other secondary outcomes were LOT, amount of different prescribed antibiotics, overall prescription rate, colitis caused by *Clostridium difficile* (CDC), infection with MRB, length of stay, detection of Respiratory Syncytial Virus (RSV) and death.

Data were analyzed with descriptive statistical methods (e.g., mean and standard deviation, SD). Results were statistically tested using IBM SPSS Statistics®. We performed the Chi Square Test for the primary outcome and Mann-Whitney tests for secondary outcomes with non-parametric data. The level of significance was adjusted using Bonferroni correction for multiple testing.

## Results

The primary outcome “adherence to treatment guidelines” increased significantly with the implementation of our ASP. The considered diagnoses for the primary outcome were AOM, pneumonia, meningitis, tonsillitis and UTI. In the pre-intervention group 100 cases with antibiotic prescriptions within the defined diseases were determined of which 33 were treated according to treatment guidelines. After the intervention 101 cases were identified in the defined period and 64 of them were treated according to the newly established SOPs (see Table 2). The *p*-value was 0.000016 in the Chi Square test.

**Table 2:** Contingency table of the primary outcome guideline-conform treatment.

	**Guideline-conform treatment**		
	**No**	**Yes**	**∑**
Before intervention	67	33	100
After intervention	37	64	101
∑	104	97	201

Guideline-conform treatment differed substantially between the most frequent diagnoses (see Table 3). Especially in the treatment of pneumonia a large increase was achieved after the intervention, from 22.8 to 64.9%.

**Table 3:** Guideline-conform treatment of the most frequent diagnoses.

	**Guideline-conform treatment**	**Before ASP (%)**	**After ASP (%)**
Pneumonia	Yes	13 (22.8%)	37 (64.9%)
	No	44 (77.2%)	20 (35.1%)
UTI	Yes	14 (66.7%)	17 (77.3%)
	No	7 (33.3%)	5 (22.7%)
Tonsillitis	Yes	4 (36.7%)	5 (50%)
	No	7 (63.6%)	5 (50%)

In total 329 patients were treated in the two observation periods and met the inclusion criteria. There were eleven cases with insufficient documentation that were not included in the analyses. Patients had a mean age of 3.2 years with a standard deviation of 4.3 years. Median age was 1.4 years with a broad variance comprising a minimum of 0 and a maximum of 17.9 years of age. Of the admitted patients, 40 of 329 had been treated with antibiotics prior to admission to the hospital. In the first quarter of 2017, 852 patients were admitted to the hospital. In this period, 164 of 852 patients were treated with antibiotics (19.2%). After the intervention, 165 of 882 patients received antibiotics (18.7%), indicating no substantial change in the rate of antibiotic treatments.

**Figure 1:**
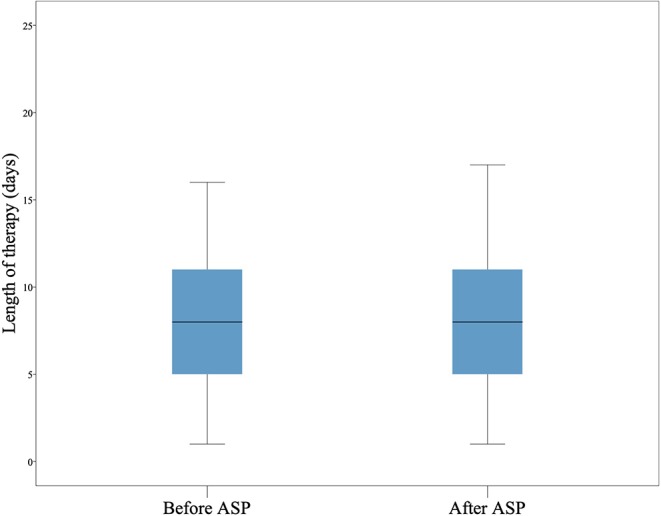
Length of therapy. The length of therapy (LOT) in days is shown as boxplot. The LOT did not differ before and after the intervention.

The use of cephalosporins decreased significantly after the intervention. Especially the use of second generation cephalosporins was reduced. Accordingly, the consumption of aminopenicillins with and without beta-lactamase inhibitors (BLI) increased significantly (see Table 4). The amount of other antibiotics did not change significantly. In some cases, the total DOT of a substance, e.g., gentamicine, was too low to draw statistical conclusions. The LOT did not differ before and after the intervention (Figure 1). Likewise, the amount of different antibiotic substances did not change significantly after the intervention. Prior to the intervention a mean of 1.7 (SD 0.8) different substances were prescribed, afterwards 1.9 (SD 0.8). No cases of CDC were observed before or after the intervention. We found two cases of MRB (3MRGN) related UTIs, both after ASP implementation. The mean length of stay changed from 4.6 to 5.5 days, respectively.

**Table 4:** Antibiotic consumption data before and after the implementation of the antibiotic stewardship program.

	**Total DOT**				**DOT per 1,000 PD**	
	**Before**	**After**	**%**	*p*-value^*^	**Before**	**After**
Cephalosporins total	**787**	**486**	**−38%**	**0.000**	**306.7**	**181.1**
Cephalosporins 1st generation	11	0	−100%	0.155	4.3	0.0
Cephalosporins 2nd generation	**638**	**257**	**−60%**	**0.000**	**248.6**	**95.8**
Cephalosporins 3rd generation	138	229	+66%	0.126	53.8	85.4
Aminopenicillins ± BLI	**240**	**529**	**+120%**	**0.000**	**93.5**	**197.2**
Aminopenicillins	136	146	+7%	0.354	53.0	54.4
Aminopenicillins + BLI	**104**	**383**	**+268%**	**0.000**	**40.5**	**142.8**
Penicillins	44	67	+52%	0.259	17.1	25.0
Piperacillin/Tazobactam	158	176	+11%	0.898	61.6	65.6
Macrolides	117	90	−23%	0.539	45.6	33.5
Lincosamides	89	88	−1%	0.852	34.7	32.8
Aminoglycosides	15	54	+260%	0.345	5.8	20.1
Glykopeptides	2	11	+450%	0.561	0.8	4.1
Fluorchinolones	12	0	−100%	0.316	4.7	0.0
Nitroimidazoles	29	8	−72%	0.456	11.3	3.0
Carbapenems	5	13	+160%	0.564	1.9	4.8
Tetracyclines	28	17	−39%	0.644	10.9	6.3
Folic-acid inhibitors	14	19	+36%	0.662	5.5	7.1

* *Adjusted level of significance 0.0025. DOT, days of therapy; PD, patient days; BLI, beta-lactamase inhibitors*.

*Bold means significant change*.

**Figure 2:**
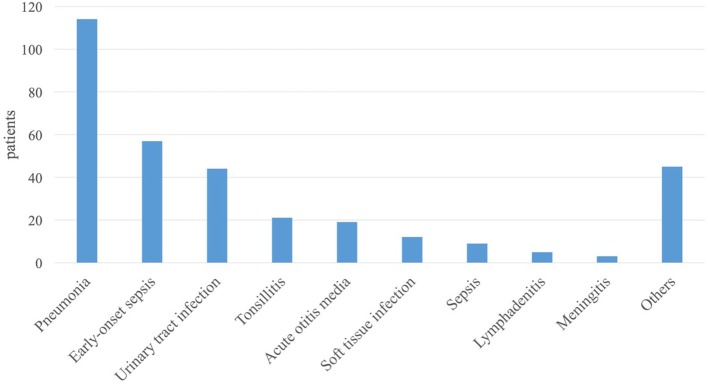
Distribution of diagnoses. The diagnoses within the study are shown in the order of frequency from left to right.

The diagnoses leading to antibiotic treatment are given in Figure 2. Pneumonia was the most frequent indication for antibiotic prescription (34.6% of all cases). Forty two of 114 cases with pneumonia were RSV positive with suspicion of bacterial superinfection. Suspected or verified early-onset sepsis (EOS) was the second most frequent indication for antibiotic treatment. However, EOS was not included for the evaluation of the primary outcome of this study as a specific in-house SOP had already been implemented for that condition prior to the start of our study. No deaths occurred in our cohort as sole recorded serious adverse event parameter of our study.

## Discussion

Here, we demonstrate effectiveness of an antibiotic stewardship program in a secondary care municipal pediatric hospital. Treatment adhering to guidelines markedly increased after our intervention and cephalosporin consumption could be replaced by aminopenicillins in many cases. Guideline adherence is one of several important items in ASPs. It was chosen as the primary outcome parameter for this study as it is unambiguously measurable and short term effects for this item were expected.

There are some data regarding sustainability of ASPs (4). In studies in which ASPs were stopped after some time, an increase of inadequate antibiotic prescriptions was observed. In our study we demonstrate an improvement of relevant outcomes 6 months after the intervention. There is still a lack and need of more long-term data to evaluate sustainability of ASPs in pediatrics. The Cochrane review by Davey et al. reported a reduction in the duration of inpatient treatment after ASP implementation. This is in contrast to our pediatric cohort and might again show the difference between adult and pediatric populations with regard to medical treatments (15).

The majority of patients in our study received antibiotic treatment due to pneumonia. In another German study investigating the effect of a pediatric ASP guideline adherence for the treatment of pneumonia was a separate outcome variable. The authors demonstrated a decrease in the use of second-generation cephalosporins from 50 to 6% (8). In line with that, we encountered a substantial reduction for cefuroxime prescriptions. The main indication for cefuroxime in our patients was severe pneumonia with hospital admission, and it was replaced by aminopenicillins with BLI after the intervention. Similar results were also obtained by other authors (8, 16, 17).

The use of third-generation cephalosporins did not change significantly. This class of antibiotics was mainly prescribed in patients with UTIs and was contained in our SOPs for this indication. Overall, the consumption of cephalosporins decreased significantly while aminopenicillin use increased. To draw clear conclusions on how replacing cephalosporins by aminopenicillins affects the prevalence of MRB has to be investigated by larger studies. The two cases of MRB-related UTIs are not sufficient to draw any conclusions. In one previous study no direct impact on the prevalence of MRB had been observed (18).

How physicians adapted the initial treatment was not evaluated in our study, nor did we evaluate the diagnostic process, which is an important part for the optimal treatment in infectious diseases. There is growing interest in the field for optimal diagnostic processes which is now often labeled as “diagnostic stewardship” (19), and we assume that there is still a large potential to improve antibiotic treatment by optimizing the diagnostic process. Especially in children there is a lack of data regarding these diagnostic processes. The overall prescription rate of antibiotics did not change substantially in our study after ASP implementation. This was similarly observed by other authors (8). Of course, diagnostic algorithms and diagnostic advice were part of the implemented SOPs and ward rounds by infectious disease experts. However, the diagnostic process was not part of our analyses which is a limitation of our study. Only patients with an antibiotic therapy were included in the study. Therefore, we can only speculate why our ASP did not result in reduction of the overall prescription rate of antibiotics. One explanation could be that the number of justified indications for antibiotics differed between the two observation periods. Another explanation could be that the indications for an antibiotic therapy were already well-standardized and restrictive in our hospital prior to the intervention.

Cost-effectiveness for pediatric ASPs was analyzed in one German and one US study at university children's hospitals with remarkable cost savings (7, 13). It remains questionable how these results can be transferred to a municipal hospital with limited use of expensive new last resort antibiotics and antifungals.

Our study is limited due to its single-center design, the relatively small patient number, and the short observation period of one quarter each in 2017 and 2018. Larger multi-center studies are warranted to confirm and possibly expand our results. Innovations in the daily workflow like electronic prescription and documentation will make it easier to evaluate ASPs in the future. Studies already showed an improvement of the prescription process in a pediatric hospital with the launch of an IT-based prescription (20). In the future, electronic real-time support and advice could assist decision-making to choose the best antibiotic therapy for each individual patient. In summary, our study shows a profound improvement in guideline-adherence after the implementation of a pediatric ASP with SOPs, prospective feedback and audits in a secondary care municipal children's hospital. Adult and pediatric ASPs might prove to be one of several elements to prevent a post-antibiotic era.

## Data Availability Statement

The datasets generated for this study are available on request to the corresponding author.

## Ethics Statement

Ethical review and approval was not required for the study on human participants in accordance with the local legislation and institutional requirements. Written informed consent from the participants' legal guardian/next of kin was not required to participate in this study in accordance with the national legislation and the institutional requirements.

## Author's Note

The work comprises part of the doctoral thesis by JM.

## Author Contributions

JM designed the study, analyzed data, and wrote the manuscript. NR designed and supervised the study and wrote the manuscript. PO and MB helped with data collection and establishment and implementation of SOPs. UB and SB assisted in study design and establishment of SOPs and reviewed the manuscript.

### Conflict of Interest

The authors declare that the research was conducted in the absence of any commercial or financial relationships that could be construed as a potential conflict of interest.
